# Antiplatelet Aggregation and Platelet Activating Factor (PAF) Receptor Antagonistic Activities of the Essential Oils of Five *Goniothalamus* Species

**DOI:** 10.3390/molecules15085124

**Published:** 2010-07-29

**Authors:** Bushra Abdulkarim Moharam, Ibrahim Jantan, Fasihuddin bin Ahmad, Juriyati Jalil

**Affiliations:** 1 Faculty of Pharmacy, Universiti Kebangsaan Malaysia, Jalan Raja Muda Abdul Aziz, Kuala Lumpur 50300, Malaysia; 2 Faculty of Resources Science and Technology, Universiti Sarawak, 94300 Kota Samarahan, Sarawak, Malaysia

**Keywords:** *Goniothalamus* species, essential oils, GC-MS, antiplatelet aggregation, platelet activating factor (PAF) antagonists

## Abstract

Nine essential oils, hydrodistilled from different parts of five *Goniothalamus* species (*G. velutinus *Airy-Shaw, *G. woodii* Merr., *G. clemensii *Ban*, G. tapis *Miq. and *G. tapisoid*es Mat Salleh) were evaluated for their ability to inhibit platelet aggregation in human whole blood using an electrical impedance method and their inhibitory effects on platelet activating factor (PAF) receptor binding with rabbit platelets using ^3^H-PAF as a ligand. The chemical composition of the oils was analyzed by gas chromatography (GC) and gas chromatography–mass spectrometry (GC–MS). The bark oil of *G. velutinus* was the most effective sample as it inhibited both arachidonic acid (AA) and ADP-induced platelet aggregation with IC_50_ values of 93.6 and 87.7 µg/mL, respectively. Among the studied oils, the bark oils of *G. clemensii*, *G. woodii*, *G. velutinus* and the root oil of *G. tapis* showed significant inhibitory effects on PAF receptor binding, with IC_50 _values ranging from 3.5 to 10.5 µg/mL. The strong PAF antagonistic activity of the active oils is related to their high contents of sesquiterpenes and sesquiterpenoids, and the individual components in the oils could possibly produce a synergistic effect in the overall antiplatelet activity of the oils.

## 1. Introduction

*Goniothalamus* (Family: Annonaceae) is a genus of about 160 species of trees and shrubs mostly found in tropical southeast Asia throughout Indochina and Malaysia [1]. In traditional medicine, different parts of the species are used to treat asthma, rheumatism, fever, malaria, cholera, stomach ache and as post-partum protective remedy, abortifacient and insect repellent [2]. Phytochemical studies on *Goniothalamus *species have resulted in the isolation of various compounds, especially styrylpyrone derivatives, quinoline and isoquinoline alkaloid derivatives and phenantrene lactones, terpenes, acetogenins and flavonoids [3,4,5,6]. Acetogenins and styryl-lactones from *Goniothalamus* species have been shown to be cytotoxic to different human tumor cell lines [7,8]. Other reported biological properties of some compounds of *Goniothalamus* species were inhibition of platelet activating factor (PAF) binding [9], antifungal [10], antiplasmodium and antimycobacterial activities [11].

Recently, the essential oils of *Goniothalamus malayanus, G. macrophyllus, G. uvariodes* and *G. andersonii* have been reported [12,13]. There is limited data on the biological properties of the essential oils of *Goniothalamus* species. A previous study [14] has demonstrated the strong larvicidal activity of *G .andersonii* oil against *Culex quinquefasciatus*, with an LC_50 _valueof 60.8 µg/mL. A recent study by Hisham *et al. * [15] showed a broad spectrum antimicrobial activity for the oil of *G. cardiopetalus*. This paper reports for the first time *in vitro* inhibitory effects of the essential oils of five *Goniothalamus *sp. viz, - *G. tapis*, *G. tapisoides*, *G.velutinus, G. woodii *and *G. clemensii* on platelet aggregation and PAF receptor binding. 

## 2. Results and Discussion

### 2.1. Chemical analysis

Water distillation of the fresh samples of *Goniothalamus * species gave various yields of oils (calculated based on dry weight, Table 1). The leaf and bark oils of *G. tapis* and *G .tapisoides* and the bark oil of *G.velutinus* may be considered satisfactory for commercial exploitation due to their high yields (>2.0%). The oils were analyzed using GC and GC/MS. The list of constituents identified in the oils is shown in order of elution on a DB-5 type column in Table 2. 

The chemical components of the essential oils of *G. tapis* and *G. tapisoides* have been previously reported by us [22]. The leaf oil of *G. tapis* was made up mainly of sesquiterpenoids with α-copaene (23.8%) and β-caryophyllene (14.4%) as the major components. The bark oil of *G. tapis* could be differentiated from its leaf oil due to its high contents of linalool (13.0%), limonene (12.7%) and safrole (11.2%). As the leaf oil, the root oil of *G. tapis* was also rich in sesquiterpenoids, but different ones were found in this oil, where the most abundant constituent was cyperene (16.2%). The leaf oil of *G. tapisoides* was comprised almost entirely of monoterpenoids, of which 1,8-cineole (79.0%) was the most abundant component. The bark and the root oils of *G. tapisoides* were also rich in 1,8-cineole, but with considerable variation in levels of the minor constituents. 

**Table 1:** Essential oil yields from different parts of five *Goniothalamus *species.

Species	Voucher No.	Part used	Yield* (%)
*G.tapis*	UM 55095	Leaf	2.23 ± 0.5
		Bark	2.85 ± 0.2
		Root	0.98 ± 0.4
*G.tapisoides*	UM 55089	Leaf	3.05 ± 0.2
		Bark	3.86 ± 0.5
		Root	1.45 ± 0.3
*G.velutinus*	UM 55097	Bark	1.40 ± 0.3
*G.clemensii*	UM 55082	Bark	2.80 ± 0.1
*G.woodii*	UM 55094	Bark	1.80 ± 0.4

* Yield based on dry weight of the respective plant parts.

The nature of the bark oils of *G. clemensii, G. velutinus* and *G. woodii* is described for the first time as they have not been reported elsewhere. Twenty three (98.1%), 45 (94.0%) and 36 (97.3%) components were identified in the bark oils of *G. clemensii, G. velutinus* and *G. woodii,* respectively. The bark oils were rich in sesquiterpene hydrocarbons and their oxygenated derivatives. A comparison between the oils of these species showed that they possessed some similarity in the composition of sesquiterpenes and sesquiterpenoids although, as one might expect, there were quantitative differences, with considerable variation in levels of the individual constituents of the oils. 

The bark oil of *G. clemensii* could be differentiated from the other oils by the absence of monoterpenes and the presence of significantly high concentrations of α-cadinol (41.6%), agarospirol (19.0%), elemol (16.1%) and seychellene (4.0%). Other compounds present in appreciable amounts in the oil were τ-muurolol (3.3%), δ-cadinene (2.7%), β-selinene (2.4%) and trans-α-bergamotene (2.0%). The major components of the bark oil of *C. woodii* were qualitatively similar to that of *C. clemensii* where α-cadinol (21.9%), elemol (12.6%), agarospirol (8.0%), cubenol (4.7%), β-caryophyllene (4.6%), τ-muurolol (4.5%), δ-cadinene (4.6%), cadalene (4.4%), γ-muurolene (3.7%), cyperene (3.7%), β-selinene (2.9%), α-humulene (2.4%), ar-curcumene (2.3%), α-copaene (2.1%) and caryophyllene oxide (2.0%) were the representatives. The bark oil of *G. velutinus *was rich in α-cadinol (14.0%), α-eudesmol (9.7%), τ-muurolol (9.1%), cubenol (7.7%), β-selinene (6.1%), γ-muurolene (5.2%) and δ-cadinene (4.7%). It could be differentiated from the other bark oils by the presence of α-eudesmol and β-bisabolol (1.5%) which were absent in the other oils. 

### 2.2. Inhibition of platelet aggregation

The antiplatelet activity of the essential oils of *Goniothalamus* species was determined in human whole blood *in vitro*. In the absence of the essential oils in the whole blood (control, DMSO 0.5%), the inducers, *i.e.,* arachidonic acid (AA), adenosine diphosphate (ADP) and collagen showed 100% platelet aggregation.

**Table 2:** Percentage composition of the essential oils of selected *Goniothalamus* species.

Components	RI a	Percentage (%)^ *^									
		*G.clemensii* (B)	*G.velutinus* (B)	*G.woodii* (B)	*G.tapis* (B)	*G.tapis* (R)	*G.tapis* (L)	*G.tapisoides* (B)	*G.tapisoides* (R)	*G.tapisoides* (L)	ID methods
Hexanal	804	-	-	-	-	-	0.3	-	-	-	a,b,c
α- Pinene	940	-	0.1	0.1	-	-	0.7	6.6	4.0	9.6	a,b,c
Camphene	953	-	-	-	-	0.3	-	-	-	-	a,b,c
Benzaldehyde	962	-	-	-	-	0.3	-	-	-	-	a,b,c
Sabinene	975	-	-	-	-	-	0.1	0.3	1.4	0.6	a,b,c
β-Pinene	985	-	-	-	-	-	-	0.1	0.1	0.9	a,b,c
Myrcene	992	-	-	-	-	-	-	1.0	0.6	-	a,b,c
α-Phellandrene	1004	-	-	-	-	-	1.0	0.1	0.1	-	a,b,c
p-Cymene	1022	-	-	-	3.8	-	1.0	-	-	-	a,b,c
p-Menthene	1027	-	-	-	-	-	-	6.9	4.2	-	a,b,c
Limonene	1029	-	-	-	12.7	-	-	-	-	-	a,b,c
β-Phellandrene	1030	-	-	-	-	-	-	t	0.1	-	a,b,c
δ-3-Carene	1031	-	-	-	1.0	-	-	-	-	-	a,b,c
Cineole	1035		0.2	0.1	-	-	7.6	47.9	56.1	79.0	a,b,c
(Z)-β-Ocimene	1038	-	-	-	-	-	-	0.1	-	-	a,b
(E)-β-Ocimene	1049	-	-	-	-	-	-	t	-	-	a,b
γ-Terpinene	1062	-	-	-	-	-	-	6.6	5.7	0.2	a,b,c
*cis*-Sabinene hydrate	1069	-	-	-	-	-	-	0.12	-	0.1	a,b
*cis*-Linalool oxide (furanoid)	1074	-	-	-	-	0.4	1.5	-	-	-	a,b
*trans*-Linalool oxide (furanoid)	1087	-	-	-	-	0.4	1.0	-	-	-	a,b
Terpinolene	1090	-	-	-	-	-	-	1.0	1.1	0.1	a,b,c
p-Cymenene	1090	-	-	-	-	-	-	0.4	0.6	t	a,b
Linalool	1109		0.22	0.8	13.0	3.9	18.5	2.3	2.2	1.0	a,b,c
α-Fenchol	1118	-	-	-	-	-	-	0.1	0.1	t	a,b
*trans*-p-2-Menth-2-en-1-ol	1121	-	-	-	-	-	-	0.1	0.4	-	a,b
*trans*-Pinocarveol	1140	-	-	-	-	-	-	-	-	0.2	a,b
Citronellal	1152	-	-	-	6.3	-	-	-	-	-	a,b,c
Borneol	1170	-	-	-	0.2	-	-	-	-	0.2	a,b,c
Terpinen-4-ol	1174	-	1.0		1.5	-	4.2	22.5	19.6	2.3	a,b,c
α-Terpineol	1187	-	0.3	0.1	-	0.1	1.8	2.8	3.2	4.4	a,b,c
Myrtenol	1191	-	-	-	-	-	-	-	-	0.1	a,b
*cis*-Piperitol	1198	-	-	-	-	-	-	0.1	0.2	-	a,b
α-Fenchyl acetate	1230	-	-	-	-	-	-	t	t	t	a,b
Linalyl acetate	1257	-	-	-	1.8	-	-	-	-	-	a,b,c
Geranial	1266	-	-	-	0.9	-	-	-	-	-	a,b,c
Safrole	1287	-	-	-	11.2	-	-	-	-	-	a,b,c
Bornyl acetate	1290	-	-	-	-	0.4	-	-	-	0.1	a,b,c
Thymol	1300	-	0.1		-	-	-	-	-	-	a,b,c
δ-Elemene	1340	-	0.1		-	0.6	-	-	-	-	a,b
Citronellyl acetate	1352	-	-	-	1.2	-	-	-	-	-	a,b,c
α-Cubebene	1354	-	-	0.1	-	0.9	1.2	t	t	0.1	a,b
Eugenol	1359	-	-	-	2.4	-	-	-	-	-	a,b,c
α-Ylanglene	1375	-	-	-	0.8	0.9	-	-	-	-	a,b
α-Copaene	1379	0.4	1.5	2.1	-	8.7	23.8	-	-	0.1	a,b,c
Geranyl acetate	1384	-	-	-	-	1.5	-	-	-	-	a,b,c
β-Cubebene	1386	-	-	-	-	1.4	-	-	-	-	a,b
α-Bourbonene	1386	0.7	-	-	-	-	3.4	-	-	-	a,b
β-Elemene	1393	0.1	0.1	0.8	-	5.3	1.1	-	-	0.1	a,b
Cyperene	1401	0.2	2.9	3.7	-	16.2	-	t	t	-	a,b
α-Gurjunene	1412	-	-	-	-	-	0.6	-	-	-	a,b
α-Cedrene	1414	-	-	-	-	0.8	-	-	-	-	a,b
β-Caryophyllene	1420	-	0.6	4.6	2.8	-	14.2	-	-	0.1	a,b,c
β-Cedrene	1422	-	-	-	-	0.4	-	-	-	-	a,b
α-Santalene	1418	0.5	0.3	0.2	-	2.5	-	-	-	-	a,b
β-Gurjunene	1429	-	-	-	-	-	0.6	-	-	-	a,b
*trans*-α-Bergamotene	1437	2.0	0.8	0.9	-	1.4	-	-	-	t	a,b
α-Guaiene	1441	-			-	1.2	-	-	-	-	a,b
Aromadendrene	1442	-	-	-	-	1.4	-	-	-	-	a,b
(Z)-β-Farnesene	1442	-	-	-	0.7	-	-	-	-	-	a,b
epi-β-Santalene	1453	-	0.6	0.2	-	-	-	-	-	-	a,b
α-Humulene	1450	0.2	0.5	2.4	1.9	-	2.5	-	-	-	a,b,c
Patchoulene	1456	-	-	-	-	1.8	-	-	-	-	a,b
Seychellene	1464	4.0	0.5	0.5	-	-	-	-	-	-	a,b
allo-Aromadendrene	1462	-	-	-	-	-	3.6	-	-	-	a,b
β-Chamigrene	1470	1.3	-	-	-	-	-	-	-	-	a,b
α-Amorphene	1474	-	1.0	-	-		-	-	-	-	a,b
γ-Muurolene	1476		5.2	3.7							a,b
Germacrene D	1479	0.2	-	1.3	-	-	-	-	-	-	a,b
ar-Curcumene	1483	-	3.9	2.3	-	-	-	-	-	-	a,b
β-Selinene	1488	2.4	6.1	2.9	-	1.9	1.6	-	-	-	a,b
α-Selinene	1493	-	-	-	-	-	0.5	-	-	-	a,b
γ-Amorphene	1498	-	-	-	-	5.2	-	-	-	-	a,b
Epizonarene	1503	-	-	-	-	2.0	-	-	-	-	a,b
α-Muurolene	1498	-	-	-	0.3	-	-	-	-	-	a,b
Eremophilene	1503	-	-	-	-	1.6	-	-	-	-	a,b
(E,E)-α-Farnesene	1505	-	-	-	-	-	0.1	-	-	-	a,b
δ-Amorphene	1512	-	-	-	-	2.5	-	-	-	-	a,b
γ- Cadinene	1513	-	0.9	-	-	2.3	0.3	-	-	0.1	a,b
δ -Cadinene	1524	2.7	4.7	4.6	0.9	3.1	0.7	-	-	t	a,b
*trans*- Calamenene	1530	-	-	-	-	1.1	0.5	-	-	0.1	a,b
Hedycaryol	1530	-	-	-	-	3.4	-	-	-	-	a,b
(Z)-Nerolidol	1533	-	-	-	0.1	0.4	-	-	-	t	a,b
*trans*-Cadia-1(2),4-diene	1536	-	-	-	-	1.9	-	-	-	-	a,b
Eudesma3,7(11)diene	1545	0.4	1.0	1.0	-	-	-	-	-	-	a,b
Elemol	1549	16.1	1.4	12.6	3.3	-	-	-	-	-	a,b
Germacrene B	1560	-	0.7	-	-	-	-	-	-	-	a,b
(-)-Spathulenol	1580	0.4	0.7	0.4	-	-	-	-	-	-	a,b
Caryophyllene oxide	1600	0.4	2.0	2.0	-	-	-	-	-	-	a,b
Globulol	1602	0.3	1.8	0.4	-	-	-	-	-	-	a,b
Guaiol	1625	-	0.7	-	-	-	-	-	-	-	a,b
Viridiflorol	1610	-	0.5	-	-	-	-	-	-	-	a,b
τ-Cadinol	1642	1.0	2.7	2.2	-	-	-	-	-	-	a,b
Cubenol	1647	-	7.7	4.7	-	-	-	-	-	-	a,b
Agarospirol	1648	19.0	2.5	8.0	4.5	1.1	-	-	-	-	a,b
τ-Muurolol	1650	3.3	9.1	4.5	-	-	-	-	-	-	a,b
β-Eudesmol	1651	-	-	-	5.6	5.0	-	-	-	-	a,b
α-Eudesmol	1655	-	9.7	-	5.8	5.7	-	-	-	-	a,b
α-Cadinol	1670	41.6	14.0	21.9	-	-	-	-	-	-	a,b
β-Bisabolol	1677	-	1.5	-	-	-	-	-	-	-	a,b
Cadalene	1679	0.8	2.1	4.4	-	-	-	-	-	-	a,b
Isolongifolen-5-one	1717	0.1	0.4	1.2	-	-	-	-	-	-	a,b
Eudesm-7(11)-en-4-ol	1705	-	1.2	1.0	-	-	-	-	-	-	a,b
(Z,Z)-Farnesol	1720	-	0.2	0.6	-	-	-	-	-	-	a,b
(E,Z)-Farnesol	1750	-	0.2		-	-	-	-	-	-	a,b
1-Octadecene	1800	-	0.3	0.6	-	-	-	-	-	-	a,b
(E,E) Farnesyl acetate	1819	-	0.3		-	-	-	-	-	-	a,b
Hexadecanoic acid	1940	-	1.6	0.1	-	-	-	-	-	-	a,b
	Total	98.1	93.9	97.0	84.5	88.0	92.4	99.0	99.7	99.4	

Percentages are expressed as peak area normalization on a DB-5 column without correction factor. t = trace, ID methods = identification methods: a = retention index, b = mass spectrum, c = co-chromatography, RI = retention index: measured relative to *n*-alkanes on the DB-5 column; R = root, B = bark, L = leaves.

The % inhibitory effects of the essential oils at 100 µg/mL were determined and those oils which showed more than 50% inhibition were further investigated at various concentrations (Table 3). Among the oils tested, the root oil of *G. tapis* and the bark oil of *G. velutinus* showed strong inhibition on platelet aggregation caused by AA, with inhibitory effects of more than 50% at 100 μg/mL. The bark oil of *G. velutinus* also showed strong inhibition on platelet aggregation induced by ADP (51% inhibition). 

**Table 3:** Percentage inhibition of *Goniothalamus* oils and aspirin on platelet aggregation of human whole blood induced by AA (0.5 mM), collagen (2 μg/mL) and ADP (10 μM).

Sample	Parts	µg/mL	AA	ADP	Collagen
*G. tapis*	Bark	100	35.34 ± 2.9	44.37 ± 4.2	23.49 ± 2.2
*G. tapis*	*R*oot	100	54.47 ± 1.2 *	46.79 ± 6.2	22.95 ± 1.7
		50	38.55 ± 0.6		
		25	25.45 ± 0.5		
		12.5	16.36 ± 0.7		
*G. tapis*	Leaf	100	18.89 ± 1.3	23.80 ± 0.7	18.29 ± 4.5
*G. tapisoides*	Bark	100	15.62 ± 1.1	37.85 ± 2.9	14.22 ± 4.1
*G. tapisoides*	Root	100	23.45 ± 2.5	46.75 ± 3.4	30.85 ± 3.6
*G. tapisoides*	Leaf	100	5.36 ± 0.0	21.46 ± 2.2	21.19 ± 3.9
*G. velutinus*	Bark	100	53.66 ± 0.3 *	51.27 ± 0.2*	32.82 ± 2.8
		50	32.27 ± 1.4	41.19 ± 0.6	
		25	17.91 ± 4.0	20.22 ±0.4	
		12.5	11.03 ± 0.3	13.81 ± 3.4	
*G. woodii*	Bark	100	42.52 ± 0.7	45.52 ± 3.7	15.27 ± 2.2
*G. clemensii*	Bark	100	43.62 ± 2.5	28.62 ± 0.7	34.52 ± 0.7
1,8-Cineol		100	22.00 ± 7.1	2.3 ± 2.6	12.25 ± 5.0
Linalool		100	22.35 ± 5.7	49.88 ± 4.8*	15.68 ± 0.6
Safrol		100	93.40 ± 5.9*	97.20 ± 1.8*	
		50	74.80 ± 0.2	74.35 ± 2.7	
		25	32.59 ± 6.9	37.15 ± 2.4	
		12.5	10.53 ± 3.7	20.34 ± 1.8	
ASA		25	99.68 ± 0.3	46.84 ± 6.2	35.19 ± 3.1
		12.5	79.41 ± 2.8		
		6.25	62.35 ± 5.3		
		3.13	51.76 ± 3.8		
		1.56	30.74 ± 0.0		

Values are presented as mean ± SE (n = 3). *p < 0.05 as compare with control. ASA = acetyl salicylic acid.

The oils showed dose-dependent responses, *i.e.,* as the concentration of the oil increased the % inhibition increased. The IC_50_ values of the active oils with mean values of three measurements are shown in Table 4. The bark oil of *G. velutinus* was an effective antiplatelet agent as it inhibited both AA and ADP-induced platelet aggregation with IC_50_ values of 93.6 and 87.7 μg/mL, respectively. The root oil of *G. tapis* showed fairly selective inhibitory activity of platelet aggregation induced by AA with an IC_50_ value of 82.3 μg/mL, but not that of other inducers. The IC_50_ values of the oils evaluated were higher than that of acetylsalicylic acid (ASA) (4.5 μg/mL or 24.8 µM), a potent cyclooxygenase inhibitor [21]. 

**Table 4:** IC_50 _values (µg/mL) of the bark oil of *Goniothalamus velutinus* and the root oil of *G. tapis*, safrole and aspirin on platelet aggregation induced by AA (0.5 mM) and ADP (10 µM) in human whole blood.

Sample	AA	ADP
*G. velutinus *(bark)	93.6 ± 3.1	87.7 ± 4.1
*G. tapis *(root)	85.3 ± 4.8	ND
Safrole	33.3 ± 6.2	28.2 ± 1.7
ASA	4.5 ± 4.5	ND

Values were calculated from at least three separate experiments. Values are represented as means ± SD. ND = not determined.

In order to correlate the chemical constituents of the oils and their antiplatelet activity, three standard compounds (linalool, cineol and safrole) that are found as major components of some oils, were also investigated for their antiplatelet effects. 1,8-Cineol, the main component of the essential oils of *G. tapisoides* and the leaf oil of *G. tapis* showed weak activity on platelet aggregation caused by all three inducers. Linalool which was present in high concentration in the essential oils of *G. tapis* showed a moderate activity (49.0%) against ADP but a weak activity against AA and collagen-induced aggregation. None of these effects was comparable to those produced by the oils themselves. However, safrole, the main component of the bark oil of *G. tapis*, showed a significant inhibitory effect against AA and ADP-induced aggregation with 93.4% and 97.2% inhibition, respectively (Table 3).

Safrole showed a dose-dependent inhibitory effect against AA and ADP-induced aggregation with IC_50_ values of 33.2 and 28.2 µg/mL, respectively. Safrole showed higher activity toward AA and ADP-induced aggregation than the oil itself. These results indicate that the antiplatelet activity of the *Goniothalamus* oils may not be due solely to any individual components but could be due to synergistic effects. A previous study has reported that the antiplatelet and antithrombotic properties *of Lavandula hybrida* oil could be due to the synergistic effect of its components [23].

### 2.3. Inhibition of PAF receptor binding

**Figure 1:**
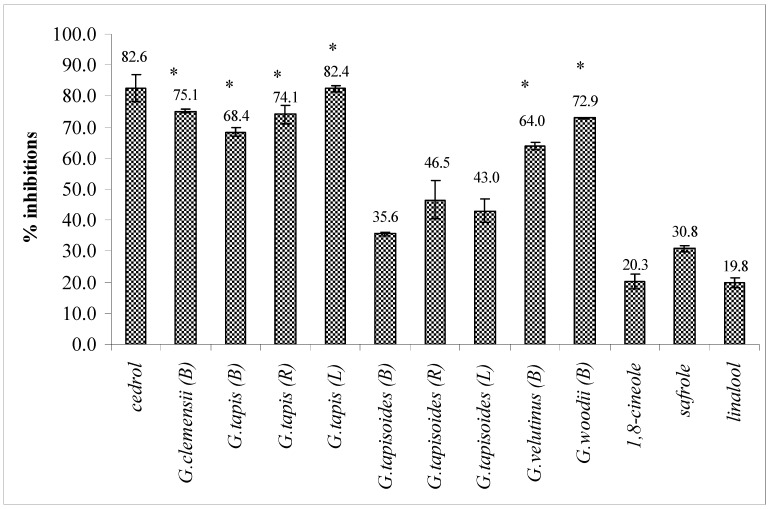
Inhibitory effects the leaf (L), bark (B) and root (R) oils of *Goniothalamus* and standards (18.2 µg/mL) on ^3^H-PAF binding to PAF receptor on rabbit platelets. Cedrol was used as a positive control. Each point represents the mean of three experiments, each in triplicate. Standard deviation of the mean are indicated as vertical bars. *p < 0.05 as compared with the respective control.

The bark oils of *G. clemensii*, *G. tapis*, *G.woodii* and *G.velutinus *and the leaf and root oils of *G. tapis* showed strong inhibitory effects on the binding of PAF to receptor on rabbit platelets at 18.2 µg/mL, exhibiting greater than 60% inhibition (Figure 1). The essential oil standards (safrole, 1,8-cineole and linalool) showed weak inhibition on the ^3^H-PAF binding. In the control group (DMSO, 0.1%), ^3^H-PAF showed 100% binding ability to the PAF receptor in rabbit platelets. Cedrol, a known PAF antagonist from natural sources [24] was used as a positive control in the bioassay. 

**Figure 2:**
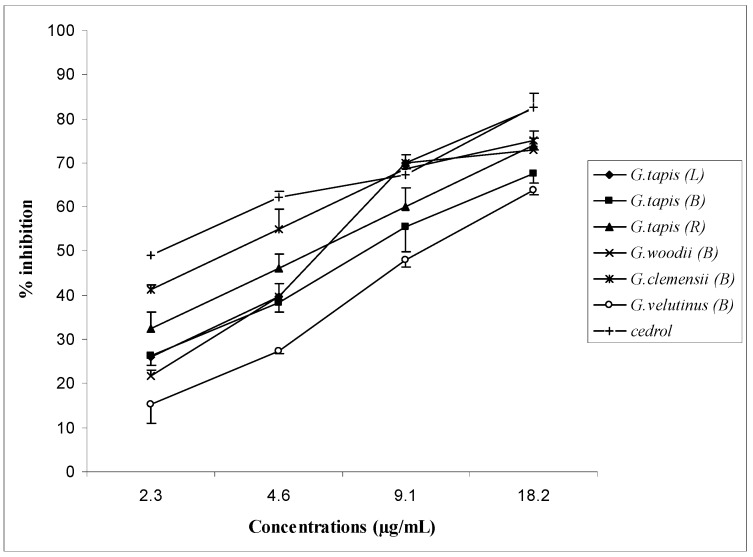
Inhibitory effect of the leaf (L), bark (B) and root (R) oils of *Goniothalamus tapis*, bark oils of *G. woodii*, *G. clemensii *and *G. velutinus* and cedrol on PAF receptor binding to rabbit platelets. Each point represents the mean of three experiments, each in triplicate. Standard deviation of the mean are indicated as vertical bars.

The percentage inhibitory effects of the active oils at various concentrations are shown in Figure 2. The oils showed dose-dependent responses, *i.e.,* as the concentration of the oil increased the percentage inhibition increased. The IC_50_ values of the active oils with the mean values of three measurements are shown in Table 5. Among the oils studied, the bark oil of *G. clemensii* was the most active, with an IC_50_ value of 3.5 µg/mL. The essential oils of *G. tapis* and the bark oils of *G. woodii* and *G. velutinus* showed comparable IC_50_ values, ranging from 5.4 to 10.5 µg/mL, but higher than that of cedrol (IC_50 _value = 2.8 µg/mL). 

**Table 5:** IC_50_ values (µg/mL) of the oils and cedrol on PAF receptor binding to rabbit platelets.

Oils	Part	IC_50_ (µg/mL)
*G. clemensii*	Bark	3.5 ± 0.2
*G. tapis*	Leaves	5.4 ± 0.2
	Bark	7.6 ± 1.2
	Root	5.5 ± 0.7
*G. woodii*	Bark	6.5 ± 0.5
*G. velutinus*	Bark	10.5 ± 1.0
Cedrol		2.8 ± 0.7

Values were calculated from at least three separate experiments. Values are represented as means ± SD.

## 3. Experimental

### 3.1. Plant material

The fresh leaves, bark and roots of *Goniothalamus tapisoides* and bark of *G.velutinus, G.woodii * and *G.clemensii* were collected from the primary forest of Sematan, Sarawak, in October 2006; while *G. tapis* was collected from the forest around Lawas in May 2006. Voucher specimens were deposited at the Herbarium of Universiti Malaysia Sarawak, Kota Samarahan, Sarawak.

### 3.2. Oil isolation and preparation of samples

The plant materials were subjected to water distillation in Clevenger-type apparatus for 6 h. The oily layers obtained were separated and dried over anhydrous magnesium sulfate. The yields were averaged over three experiments and calculated based on dry weight of the plant materials. The oils were each dissolved in dimethyl sulfoxide (DMSO) (1 mg/50 µL). The stock solutions (10 µL) were diluted in normal saline to give final concentrations of 200, 100, 50 and 25 μg/mL.

### 3.3. GC and GC-MS analyses

Gas chromatography (GC) and gas chromatography-mass spectrometry (GC-MS) analyses were used for the identification of the essential oil components. The oils were determined using a Shimadzu GC-2010 instrument equipped with a flame ionization detector (FID) and a DB-5 (30 m × 0.25 mm, 1 µm film thickness) capillary column. One µL of each sample, dissolved in ethyl acetate, was injected automatically in spilt mode (Shimadzu AOC-20i auto-injector), using pressure-controlled nitrogen as a carrier gas at a linear velocity of 50 cm^3^/min. The temperature of injector and detector was maintained at 250 ºC. The oven temperature was programmed from 75 ºC for 10 min, then at 3 ºC/min to 250 ºC and held for 5 min. The oils were also examined using stationary phase SE-30 (30 m × 0.25 mm, 0.25 µm film thickness) under the following program condition; initial temperature 60 ºC for 10 min, then 3 ºC/min to 230 ºC for 1 min. Peak areas and retention times were measured by computerized integration. The relative amounts of individual components were calculated based on the peak areas obtained without a flame ionization detector (FID) response factor correction. The linear retention indices of the components relative to n-alkanes were also determined. The oils were also analyzed using a Hewlett Packard GC-MSD 5890 series II. EI electron impact mode with electron energy 70 eV, scan time 1.5 s, mass range 40-500 Da, using a BPX5 (25 m × 0.25 mm × 0.25 µm film thickness) capillary column. Similar condition was used as described in GC programs. Identification of components was by comparing their relative retention indices with those in the literature and their mass spectral data with the existing Wiley library and co-chromatography of some components with authentic components on the DB-5 capillary column [16]. 

### 3.4. Platelet aggregation assay

Collagen, ADP, and AA were products of Chrono-Log Corp. (Havertown, PA, USA). The antiplatelet activity was performed as described by Jantan *et al.* [18]. The use of human blood was approved by the Ethics Committee of Universiti Kebangsaan Malaysia (UKM) (approval no. FF-120-2007). Briefly, blood was taken by venipuncture from healthy human volunteers based on the criteria that that they were non-smokers and had not taken any medications, including aspirin, within the last two weeks, and had not taken any food within the last 8 h. Five µL of each oil or the essential oil standard (safrole, 1,8-cineole and linalool, 20 µg/µL, in DMSO) was added followed by the inducers collagen (2 µg/mL), ADP (10 µM) or AA (0.5 mM). The final concentrations of the sample in the mixture were 100, 50, 25 and 12.5 μg/mL. The platelet aggregation was measured by a Whole Blood Lumi-Aggregometer (Chrono-Log Corp., Havertown, PA) using an electrical impedance method [19]. The mean platelet aggregation in whole blood was measured as a change in impedance over 6 min after the addition of the inducers by comparison to that of a control group impedance [20]. A mixture containing 0.5% DMSO in the diluted whole blood was used as control. Aspirin, a potent cyclooxygenase inhibitor, was used as a positive control in the bioassay [21]. The final concentration of DMSO in the whole blood was 0.5% to eliminate the effect of the solvent on the aggregation [18]. 

### 3.5. PAF receptor binding activity

Radiolabeled PAF (1-O-[^3^H]-octadecyl-2-acetyl-sn-glycero-3-phosphocholine was purchased from American Radiolabled Chemicals, (St. Louis, MO, USA) with a specific activity of 60 Ci/mmol soluble in ethanol/toluene (1:1). Unlabeled PAF and cedrol were obtained from Sigma Chemical Co. (St. Louis, MO, USA). The assay was carried out according to the modified method of Valone *et al.* [17]. The use of rabbit blood was approved by the Animal Ethical Committee of UKM (approval no. FSKB/2007/Juriyati/10-July/192). The reaction mixture consisted of 200 μL of washed rabbit platelet suspension, 25 μL of ^3^H-PAF (2.0 nM) with or without 25 μL unlabelled PAF (2.0 µM) and 25 μL of the oil, essential oil standard (safrole, 1,8-cineole and linalool) or control solution. The final concentrations of the oil sample or standard in the reaction mixtures were 18.2, 9.1, 4.5, 2.3 μg/mL. The final concentration of DMSO (control) in the reaction mixture was fixed at 0.1% to avoid interference with the receptor binding studies. The reaction mixture with 0.1% DMSO in saline was used as a control and cedrol was used as a positive control. The reaction mixture was incubated at room temperature for 1 h. The free and bound ligands were separated by filtration technique using Whatman GF/C glass fibre filters. The radioactivity was measured by scintillation counter (Packard Tri-Carb, models 2100TR/2300TR, Germany). The difference between the total amounts of ^3^H-PAF bound in the absence and in the presence of excess unlabelled PAF was defined as specific binding of ^3^H-PAF. The specific binding is expressed is expressed as percent inhibition of the control. The IC_50_ values of the samples were obtained from at least three independent determinations. 

### 3.6. Statistical analysis

All the data were analysed using *Statistical Package for Social Sciences* (SPSS) version 15.0. Each sample was measured in triplicate and the data are presented as means ± standard deviation (SD). Probit programme was used to determine the IC_50_ value for active extract. The values were obtained from at least three determinations. Data were analysed using a one way analysis of variance (ANOVA) for multiple comparisons. p < 0.05 was considered to be statistically significant. 

## 4. Conclusions

A chemical composition-PAF antagonistic activity analysis demonstrates that the strong PAF antagonistic activity of the active *Goniothalamus* sp. oils is related to their high contents of sesquiterpenes and sesquiterpenoids, although other constituents may also contribute to the activity of the oils (Table 2 and Figure 1). It is difficult to correlate the activity of any oil with its individual components as these components could possibly produce a synergistic effect on the overall PAF antagonistic activity of the oils. The inhibitory activity may be due to the different modes of action of the total components of the oils towards PAF receptor binding. Further studies need to be carried out to identify the active compounds in *Goniothalamus* oils and to find the lead structure with maximum inhibitory activity.
